# Child Development and Molecular Genetics: 14 Years Later

**DOI:** 10.1111/j.1467-8624.2012.01757.x

**Published:** 2012-03-30

**Authors:** Robert Plomin

**Affiliations:** King’s College London

## Abstract

Fourteen years ago, the first article on molecular genetics was published in this journal: *Child Development, Molecular Genetics*, *andWhat to Do With Genes Once They Are Found* (R. Plomin & M. Rutter, 1998). The goal of the article was to outline what developmentalists can do with genes once they are found. These new directions for developmental research are still relevant today. The problem lies with the phrase “once they are found”: It has been much more difficult than expected to identify genes responsible for the heritability of complex traits and common disorders, the so-called *missing heritability* problem. The present article considers reasons for the missing heritability problem and possible solutions.

The first article on molecular genetics in *Child Development* was published in 1998 with the title: “Child Development, Molecular Genetics, and What to Do With Genes Once They Are Found” (Plomin & Rutter, 1998). The article considered three general topics: finding genes (how DNA variants are used to find linkages and associations between genes and behavior), getting genes (the practicalities of how developmentalists can get DNA and genotype their samples for specific genes), and using genes. This last topic, using genes, was the main point of the article.

Where are we now, 14 years later? The basic descriptions of the first two topics—finding genes and getting genes—remain relevant although some updating is necessary. For example, in relation to finding genes, the field is now dominated by a technique that was not possible in 1998 (genome-wide association [GWA]). In terms of getting genes, the major technology used today was not invented in 1998 (microarrays), and the major technology that is beginning to revolutionize this field would have been thought to be wishful thinking just a few years ago (whole-genome sequencing). This article updates the 1998 article in relation to these three advances.

The examples of the key third topic—using genes—are just as relevant today: answering questions about developmental continuities, about psychopathological patterns, and about environmental risk mechanisms. These examples of “what to do with genes once they are found” led us to encourage developmentalists to obtain DNA in their research—not to join in the hunt for genes but to be able to use these genes once they are found. All would be well now 14 years later except for one crucial problem: Progress has been slow in finding genes associated with behavior. This problem is the focus of the present article, which discusses reasons why progress has been slow, attempts to address the problem, and implications for developmentalists.

The 1998 article was overly optimistic, especially in the use of the word “soon” in the following quote: “Although gene-behavior associations are currently available in only a few domains, the intensity of the research effort to find genes associated with behavior makes it likely that such associations will soon be widespread” (Plomin & Rutter, 1998, p. 1225). The article concluded: “We have no doubt that this potential, when actualized, will transform both developmental psychology and developmental psychopathology” (p. 1223).

I continue to support this view, even though it will take more time for this potential to be actualized. Although the 1998 article was overly optimistic about the time it would take, it was merely obeying what has been called “the first law of technology” by Francis Collins in his book on the DNA revolution: “The consequences of a radical new technology are almost always overestimated in the short term and underestimated in the long term” (Collins, 2010).

The first section of the present article describes three major technological advances since 1998: whole-genome sequencing, microarrays, and GWA. The second section reviews the first harvests from GWA studies, which leads to what has been called the missing heritability problem. The final section considers ways in which the missing heritability problem is being addressed and its implications for developmental research.

## Since 1998: Whole-Genome Sequencing, Microarrays, and GWA

During the past 14 years, there have been many advances in the technology-led field of molecular genetics, but three are especially worth noting because they will have a direct impact on developmentalists: whole-genome sequencing, microarrays, and GWA.

### Whole-Genome Sequencing

The most fundamental breakthrough since 1998 has been identifying the sequence of the 3 billion nucleotide base pairs across the 23 pairs of chromosomes in the human genome. The Human Genome Project was completed in 2003, exactly 50 years after the discovery of the structure and function of DNA (Watson & Crick, 1953). It required the effort of 2,000 researchers and cost $3 billion (http://www.genome.gov/11006943). As an indication of the speed of technological innovation in molecular genetics, it is now possible to sequence the genome of an individual in a few hours for less than $20,000 in high-throughput DNA factories with hundreds of sequencing machines and technicians. Eight groups are now closing in on the $10 million Archon X Prize for a so-called “next generation sequencing” device that can completely sequence 100 human genomes within 10 days at a cost of no more than $10,000 per genome (http://genomics.xprize.org/).

There are two major ways in which whole-genome sequencing will affect developmentalists. The first impact is conceptual. Prior to the Human Genome Project, research focused on the less than 2% of DNA, that constitutes what has traditionally been called a gene—that is, DNA that is transcribed into RNA, which is then translated into the amino acid sequences that are the building blocks of proteins. About 20,000 such “genes” have been identified in the human genome. The remaining 98% of the genome was thought to be evolutionary detritus. However, the Human Genome Project opened up a new world of genetics, leading to research that shows that at least half of the rest of the genome involves DNA that is transcribed into RNA but the RNA is not translated into amino acid sequences. For these so-called noncoding genes, the RNA itself regulates the expression of other genes, provoking thought about what a gene is (Gerstein et al., 2007). Although many of the thousands of rare single-gene human disorders involve mutations in coding genes, noncoding genes may contribute more subtly to the heritability of the complex traits and common disorders that are the focus of the behavioral sciences. Information from the whole genome, not just the 2% of DNA involved in coding genes, is needed to investigate noncoding genes as well as other new discoveries about the genome.

The second way in which whole-genome sequencing will affect developmentalists is more practical but highly contentious. As the cost of sequencing the genome continues to plummet, the entire sequence of the genome will be known for many individuals. In 2008, the 1000 Genomes Project was launched, which will catalog human genetic variation throughout the world (Durbin et al., 2010). In 2010, a 10,000 Genomes Project was funded by the Wellcome Trust in the United Kingdom, which will identify even rarer DNA variants (http://www.wellcome.ac.uk/News/Media-office/Press-releases/2010/WTX060061.htm). Some members of the genomics community have predicted that whole-genome sequences will be a standard part of medicine in the next few years. For example, Francis Collins, the director of the U.S. National Institutes of Health and former director of the Human Genome Project, after describing caveats and cautions, predicts in his book on “personalized medicine”: “I am almost certain, however, that whole-genome sequencing will become part of newborn screening in the next few years. . . . It is likely that within a few decades people will look back on our current circumstance with a sense of disbelief that we screened for so few conditions” (Collins, 2010, p. 50). The experience of having his genome sequenced has been described by psychologist Steve Pinker (2009), who was one of the first 10 people to have their genome sequenced as part of the Personal Genomes Project, which aims to give everyone access to their whole-genome sequence for personalized medical decisions (http://www.personalgenomes.org).

There are many issues involved in this DNA revolution (Guttmacher, McGuire, Ponder, & Stefansson, 2010) but the simple point here is that if whole-genome sequence data becomes part of newborn screening or is otherwise widely available for many individuals, no DNA would need to be collected and no genotyping would need to be done in order to make use of this genome sequence data in developmental research.

### DNA Microarrays

Because it is not clear when whole-genome sequencing will be widely available, developmental researchers will first use DNA microarrays that can currently genotype 1 million DNA variants across the genome on a microarray or “chip” the size of a postage stamp (Plomin & Schalkwyk, 2007). Of the 3 billion nucleotide base pairs of DNA in the human genome, fewer than 0.5% (i.e., 5 in 1,000) differ for at least 1% of the population, although there are many more rarer variants seen in fewer than 1% of the population. Because only DNA differences can account for heritability, an interim strategy is to focus on DNA variants rather than sequencing the entire genome. Although it has been possible for decades to detect DNA variants such as SNPs, genotyping them one by one is very expensive in time and money. Microarrays, which became available commercially in 2000, can genotype millions of SNPs in parallel quickly and inexpensively. A microarray is a tiny slide that is dotted with short single-stranded DNA sequences called probes. The microarray is used to detect SNPs in the usual way: Fluorescently labeled single-stranded DNA from an individual is allowed to hybridize with a single-stranded probe which will only happen if there is an exact match of DNA sequence. The critical difference with microarray analysis is that an individual’s entire genome is first chopped into small DNA pieces and all the pieces are copied many times; a florescent tag is attached to each DNA piece so that florescence will be detected if the piece of DNA hybridizes with a probe on the microarray. This method makes it possible to genotype all the DNA pieces simultaneously. Multiple copies of each target on an array make the genotyping highly accurate. Microarrays can also be used to study expression levels of all the genes in the genome, called the transcriptome. Such RNA microarrays assess the quantity of all the RNA transcripts in the genome, which is specific to tissue, age, and state. Although we mention gene expression later, our focus here is on DNA sequence variation, which is not tissue, age, or state specific.

DNA microarray platforms can genotype millions of SNPs selected to capture as much DNA variation as possible throughout the genome. These microarrays and their progenitors were designed to facilitate GWA, which is discussed in the following section. However, any DNA probes can be selected for genotyping on a microarray. For example, microarrays could include rare SNPs rather the common SNPs typically used on microarrays designed to maximize genomic information for GWA; we will return to this issue in the later section on “missing heritability.”

Some microarrays include probes for a type of DNA variant called a copy number variant (CNV), which was discovered as a result of the Human Genome Project because these regions of the genome were difficult to sequence. CNVs are DNA segments from a thousand to millions of bases in size that are deleted or duplicated, which can lead to the duplication or deletion of whole genes or parts of them (Conrad et al., 2010; Cooper, Zerr, Kidd, Eichler, & Nickerson, 2008; Redon et al., 2006). CNVs are also part of the story of missing heritability, as discussed later.

Microarrays can be customized to genotype DNA variants for disease, which is the source of the burgeoning business of direct-to-consumer genetic testing, such as the service offered by 23andMe which tests for over 100 diseases and traits for about $100. Again there are huge issues to consider including issues of practical utility, but it is noteworthy that in his book on the DNA revolution in personalized medicine, Collins (2010) is a proponent of direct-to-consumer genetic testing.

Customized DNA microarrays are quickly becoming more specialized. For example, specialized DNA microarrays are now available for all DNA variants related to cardiovascular (CardioChip) and immunological (ImmunoChip) function and dysfunction. The cost of creating a customized DNA microarray is substantial but the cost of using such a microarray drops as a function of the quantity produced. For example, a developmental DNA microarray (DevoChip?) could be customized for domains of behavioral development that include all of the many hundreds or thousands of DNA variants that may be associated with each domain at different ages as they interact and correlate with different environments. The ability to genotype thousands of DNA variants cheaply is crucial because, as discussed later, the heritability of complex traits and common disorders is due to many genes. Individually, these genes have such small effects in the population that they will be of little use to developmentalists. However, together in polygenic composites, they could have a major impact on developmental research, as discussed later.

### Genome-Wide Association

As mentioned in the previous section, DNA microarrays, with their ability to genotype millions of DNA variants quickly and cheaply, made it possible to look for DNA associations systematically throughout the entire genome, called GWA. For decades, linkage had been used as a genome-wide approach that required only a few hundred DNA variants to identify the genes responsible for hundreds of single-gene disorders such as Huntington’s disease, phenylketonuria, and fragile-X syndrome. Linkage looks for coinheritance of a DNA variant and a disorder within families (i.e., a violation of Mendel’s second law of independent assortment), as explained in the 1998 article. Coinheritance of the DNA variant and the disorder indicates that they are “linked” on the same chromosome; the tighter the coinheritance, the closer the DNA variant and gene for the disorder are on the chromosome.

Although linkage works very well for finding genes responsible for monogenic disorders, it lacks power to detect genes of smaller effect size (Risch & Merikangas, 1996). It became apparent in the 1990s that linkage studies were largely coming up empty-handed in research on common disorders, which suggested that the heritability of these phenotypes is not due to a single gene. The power of linkage methods was increased by studying many small family groups (e.g., parent–offspring triads or siblings) rather than a few large family pedigrees, but linkage could still only detect relatively large effects and few of these were found for common disorders.

While linkage is systematic but not powerful, association is powerful but, until the advent of microarrays, association was not systematic. Association involves a correlation between an allele and a trait among unrelated individuals in a population in the sense that individuals with a particular allele differ on the trait from individuals with different alleles. Association is much more powerful statistically than linkage for identifying genes of small effect size (Risch & Merikangas, 1996).

The advantage of linkage is that it is far sighted in the sense that linkage can be detected when the DNA variant is millions of base pairs away from the causal DNA variant, which is why only a few hundred DNA markers are needed to detect linkage across the genome. In contrast, association is near sighted in that it detects association only when the DNA variant is within a few thousand base pairs of the causal DNA variant, thus requiring hundreds of thousands of DNA markers to detect association across the genome.

For this reason, until microarrays became available, association studies focused on specific candidate genes, that is, genes that have some rationale for being associated with the trait. A few candidate genes such as dopamine and serotonin genes have been used in hundreds of behavioral studies, including developmental studies, during the past two decades. One problem with the candidate gene approach is that we often do not have strong hypotheses as to which genes are candidate genes. Indeed, the general rule of pleiotropy (each gene has many effects) suggests that most of the thousands of genes expressed in the brain could be considered as candidates. Another problem is that candidate gene studies are limited to the 2% of the DNA in traditional coding regions, as mentioned earlier.

However, the biggest problem for candidate gene association studies was that their results were difficult to replicate (Tabor, Risch, & Myers, 2002). This was a general problem for all complex traits, not just for behavior (Ioannidis, Ntzani, Trikalinos, & Contopoulos-Ioannidis, 2001). For example, in a review of 600 reported associations with common medical diseases, only 6 had been consistently replicated (Hirschhorn, Lohmueller, Byrne, & Hirschhorn, 2002), although a follow-up meta-analysis indicated greater replication for larger studies (Lohmueller, Pearce, Pike, Lander, & Hirschhorn, 2003). As discussed next, the reason for these failures to replicate was primarily that the largest effect sizes are much smaller than expected and the studies were underpowered to detect them. Few candidate gene associations have been replicated in GWA studies (Siontis, Patsopoulos, & Ioannidis, 2010).

Since 2005, GWA has revolutionized attempts to find DNA variation responsible for the heritability of common disorders and quantitative traits (Hirschhorn & Daly, 2005). The genome-wide feature of GWA refers to the genotyping of hundreds of thousands of common DNA variants distributed throughout the genome that “tag” most of the common sequence variation in the genome. That is, with a million well-chosen SNPs, adjacent SNPs on a chromosome correlate greater than 0.95, so that SNPs in between these pairs of SNPs add little additional information. GWA is hypothesis free in the sense that it is not limited to candidate genes, nor is it limited to DNA in coding regions. This hypothesis-free aspect of GWA is important because new sources of DNA variation continue to be discovered, such as CNVs and noncoding RNA mentioned earlier. More than 80% of associations found in GWA studies fall outside coding regions (Manolio et al., 2009). GWA was first proposed in 1996 (Risch & Merikangas, 1996), but because microarrays were not yet commercially available, the only attempts to conduct GWA involved painstaking and expensive genotyping of each DNA marker individually, which meant that only a few thousand markers were genotyped (e.g., Plomin et al., 2001). Two articles propelled GWA forward. The first was published in 2005, using GWA to identify an association of large effect for age-related macular degeneration (Klein et al., 2005). Although the GWA sample was very small by current standards (96 cases, 50 controls) and the first commercially available microarrays included only 100,000 SNPs, an association was identified with an odds ratio of nearly 5 and a population attributable risk of about 50%. The frequency of the risk allele was 72% in cases and 36% in controls. The association was quickly accepted for three related reasons: It was a large effect, it was immediately replicated in other independent studies (Thakkinstian et al., 2006), and it was in an unexpected gene (complement factor H) but one that made sense in retrospect and that opened up new therapeutic possibilities. Moreover, this success was quickly followed by finding another gene associated with macular degeneration that also had a large effect (Dewan et al., 2006) and was robustly replicated (Chen et al., 2009). Most of the genetic risk for age-related macular degeneration could be predicted by these two genes. These results electrified the scientific community. The results were especially exciting because both genes involved inflammatory pathways which had not been considered as important in relation to the disorder. Research along these lines has led to clinical trials that are now underway to test the efficacy of anti-inflammatory agents to prevent macular degeneration (Collins, 2010).

This initial GWA success was followed by several GWA articles reporting much smaller associations, such as an association between SNPs in the interleukin-23 gene and inflammatory bowel disease, which showed an odds ratio of about 1.5 between cases and controls (Duerr et al., 2006), a finding replicated in several subsequent studies (Cho & Weaver, 2007). The initial surge of GWA research culminated in 2007 with the groundbreaking Wellcome Trust Case Control Consortium (WTCCC) article that reported GWA results for 500,000 SNPs genotyped for seven common disorders each with 2,000 cases and 3,000 shared controls in collaboration with 50 research teams (Wellcome Trust Case Control Consortium, 2007). More than 20 associations across the seven disorders were significant despite correction for the massive multiple testing involved in GWA (*p* = 5 × 10^−7^). Bipolar disorder, the only behavioral disorder, and hypertension showed weaker results than the other disorders, with associations only reaching “suggestive” significance (*p* = 5 × 10^−5^).

The journal *Science,* declared GWA the “Breakthrough of the Year” for 2007. As of May 2011, 896 independent GWA published articles claimed genome-wide significant associations for 4,459 SNPs (http://www.genome.gov/gwastudies/).

## The Missing Heritability Problem

Although the first GWA success with macular degeneration discovered two genes of large effect, subsequent GWA studies have found much smaller effects. Using the WTCCC results as an example, the largest association for bipolar disorder was based on the small allele frequency difference of 47% in cases versus 43% in controls, an odds ratio of just 1.2. One of the most significant results, the association on chromosome 9 with coronary heart disease, yielded an odds ratio of only 1.4. The only two large effect sizes were for rheumatoid arthritis and Type 1 diabetes; both involved the major histocompatibility locus on chromosome 6, which was already known to harbor many genes associated with these and other disorders. Despite its large samples, WTCCC was only powered to detect effect sizes larger than the effect sizes that were actually detected, which has three implications. First, some of these associations seem likely to fail to replicate in independent samples. Second, even larger sample sizes and more powerful methodologies are needed to detect such small effects. The third implication is that associations of large effect size can be excluded. That is, although WTCCC had little power to detect small associations, it had great power to detect larger effect sizes and failed to do so.

The WTCCC results are typical of the hundreds of subsequent reports on GWA analyses of common disorders and quantitative traits, including behavioral disorders and traits (Hindorff, Junkins, Hall, Mehta, & Manolio, 2010; http://www.genome.gov/gwastudies/). The largest odds ratios from GWA studies of cases and controls are typically less than 1.2 (McCarthy et al., 2008). For quantitative traits, one of the largest effect sizes (1%) is for the association between the FTO gene and body weight (Frayling et al., 2007), a highly replicated finding (Walley, Asher, & Froguel, 2009). The largest effect sizes for height are even smaller (Lango Allen et al., 2010). For behavioral traits, the largest effect sizes in the first GWA studies of reading, mathematics and general cognitive ability assessed as quantitative traits in children are less than 0.5% of the variance (Butcher, Davis, Craig, & Plomin, 2008; Docherty, Davis, et al., 2010; Meaburn, Harlaar, Craig, Schalkwyk, & Plomin, 2008).

If the largest associations are so small, it is a safe prediction that the tail of the distribution of effect sizes will involve effects that are infinitesimally small, perhaps “private” mutations that are idiosyncratic to each individual. Hundreds or even thousands of such associations will be needed to explain the heritabilities of behavioral traits, which are typically about 50% (Plomin, DeFries, McClearn, & McGuffin, 2008). So far, putting all known SNP associations for any trait explains only a small proportion of heritability, typically about 5% (Manolio et al., 2009). An analysis based on existing GWA findings for several traits suggests that at most 20% of the known heritability of these traits can be detected (Park et al., 2010). For the GWA studies of cognitive development just mentioned, composites of the top hits accounted for less than 5% of the variance of these traits.

The most informative data come from studies of height, which is 90% heritable, in contrast to typical heritabilities of 50% for behavioral traits. At least 40 genes were found to be associated with height from the first wave of GWA studies, but together they account for only about 5% of the variance of height in studies of tens of thousands of people (Visscher, 2008). A more recent study of 183,000 individuals indicates that with 180 variants it is possible to explain about 10% of the variance (Lango Allen et al., 2010), although it has been suggested that much more of the heritability of height could be accounted for using SNPs on existing microarrays given sufficiently large samples (Visscher, Yang, & Goddard, 2010; Yang et al., 2010). Similarly for weight, a two-stage meta-analysis of 250,000 individuals identified 32 SNPs that together explain about 10% of the heritability of body mass index (Speliotes et al., 2010).

This gap between GWA-identified associations and heritability has become known as the missing heritability problem (Maher, 2008). Can the largest effects really be so small? Gene hunters are still recovering from the shock of finding that the largest associations account for so little variance in the population—after all, it was only two decades ago that the field was limited to detecting monogenic effects that account for all of the heritability.

## Finding the Missing Heritability

The missing heritability problem needs to be remedied or at least relieved before the potential of DNA can be actualized in developmental research. How much of the heritability needs to be accounted for before DNA can be useful for developmental research depends on the research question. Although it seems unlikely that all of the missing heritability will be found, for reasons discussed later in this section, GWA research might eventually identify more than half of the heritability. This criterion fits with another criterion for GWA success: to exceed the prediction from family data (Aulchenko et al., 2009). First-degree relatives are on average 50% similar for additive genetic effects so that identifying half of the heritability should exceed the prediction that is possible from first-degree relatives. However, DNA predictions can be more valuable than family risk estimates for three reasons. First, DNA predictions are specific to individuals within a family in contrast to family risk estimates, which are the same for all members of a family. Second, predictions based on DNA are limited to genetics whereas predictions from family risk can include nurture as well as nature. Third, DNA sequence variation does not change during development whereas family risk estimates—for example, using parents’ characteristics to predict children’s risks—can be complicated by developmental change.

For some research questions, predicting even 5% let alone 50% of the heritability could be useful. As mentioned earlier, it is possible to aggregate the small effects of many DNA variants associated with a trait (Wray, Goddard, & Visscher, 2008). Such polygenic composites have been called polygenic susceptibility scores (Pharoah et al., 2002), genomic profiles (Khoury, Yang, Gwinn, Little, & Flanders, 2004), SNP sets (Harlaar, Butcher, Meaburn, Craig, & Plomin, 2005), genetic risk scores (Morrison et al., 2007), and aggregate risk scores (Purcell et al., 2009). Polygenic composites are beginning to be used to predict the population-wide genetic risk for common disorders, such as breast cancer (Pharoah et al., 2007), atherosclerosis (Morrison et al., 2007), coronary heart disease (Bare et al., 2007), age-related macular degeneration (Maller et al., 2006), recurrent venous thrombosis (Vljeg, Baglin, Bare, Rosendaal, & Baglin, 2008), and Type 2 diabetes (Lyssenko et al., 2005), although the practical limitations of these approaches are increasingly recognized (e.g., Paynter et al., 2010). For quantitative traits, height is an exemplar for research using polygenic composites (Lango Allen et al., 2010; Visscher, 2008; Visscher et al., 2010; Yang et al., 2010).

The GWA research on learning and cognitive abilities in children mentioned earlier provides empirical examples. A polygenic composite (“SNP set”) based on fewer than a dozen SNPs, each of which accounted for less than 0.5% of the variance, yielded a normal distribution of genetic liability. Even though the polygenic composites only explained 3%–5% of the total variance, the top and bottom 10% of these genotypic distributions differed by 1 *SD* on the measures of cognitive development (Butcher et al., 2008; Davis et al., 2010; Docherty, Davis, et al., 2010; Meaburn et al., 2008). These SNP sets have been used to investigate issues such as early prediction, multivariate issues, and issues of genotype–environment interplay (Docherty, Kovas, Petrill, & Plomin, 2010; Harlaar et al., 2005; Haworth, Meaburn, Harlaar, & Plomin, 2007). In addition, reliable polygenic composites of this magnitude could be useful for genotypic selection of groups at low and high genetic risk in areas of research such as neuroimaging where large samples are difficult to study.

Dozens of articles have been published about the missing heritability problem and ways to solve the problem, although as yet there is no consensus (e.g., Eichler et al., 2010; Frazer, Murray, Schork, & Topol, 2009; Ku, Loy, Pawitan, & Chia, 2010; Manolio et al., 2009; McCarthy et al., 2008). We begin by discussing heritability itself.

### Has Heritability Been Overestimated?

One easy explanation of the missing heritability problem is that heritability might have been overestimated. Heritability is a statistic that estimates the extent to which observed (phenotypic) individual differences for a trait can be accounted for by genetic differences among individuals in a particular population with its particular mix of genetic and environmental differences at the time of assessment. Heritability is a descriptive population statistic that will change as genetic and environmental influences change in the population. It refers to differences among individuals, not to a single individual for whom both genotype and environment are indispensable (Vineis & Pearce, 2010). Heritability does not imply genetic determinism. Interpretations and misinterpretations of heritability have been discussed elsewhere (e.g., Plomin et al., 2008; Rutter, 2006; Visscher, Hill, & Wray, 2008).

Because the source of heritability is inherited differences in DNA sequence, the ultimate test of the accuracy of heritability estimates is the identification of all of the DNA sequence variation responsible for heritability. Until then, it remains a possibility that heritability has been overestimated. One reason to think that heritability estimates are roughly accurate is that the basic quantitative genetic designs used to estimate heritability—family, adoption and twin designs—generally converge on similar estimates of heritability. Each of the designs has potential problems, but they have different problems, which makes this convergence reassuring. This convergence has been overlooked in some recent discussions about heritability and molecular genetics. For example, it has been suggested that monozygotic (MZ) twins could share newly appearing (de novo) mutations of large effect such as CNVs that are not shared by dizygotic (DZ) twins, and this could inflate estimates of heritability (Clarke & Cooper, 2010). However, this speculation is difficult to square with the converging results from family and adoption studies of first-degree relatives. Moreover, GWA data generally confirm the basic premise of the twin method that MZ twins are genetically identical and DZ twins are 50% similar on average (Visscher et al., 2006).

It should be noted that even if heritability has been overestimated by a factor of two—that is, heritability is 25% instead of 50%—there would still be a missing heritability problem because known associations to date account for less than 5% of the variance of common disorders and quantitative traits.

### Common Variants Could Have Very Small Effects

It is generally accepted that there is a missing heritability problem. The main strategy so far to address the problem is to continue to use commercially available microarrays, which only assess common variants, but to increase sample sizes, often through meta-analyses of results from several studies, in order to detect smaller effects. This approach has led to some successes, for example, for disorders such as schizophrenia (Purcell et al., 2009; Shi et al., 2009; Stefansson et al., 2009; Stone et al., 2008), autism (Weiss et al., 2008), Type 2 diabetes (Zeggini et al., 2008), Crohn’s disease (Barrett et al., 2008), as well as quantitative traits such as height (Weedon et al., 2008) and weight (Willer et al., 2009). However, these studies underline the conclusion that the largest effects of common variants are very small, with odds ratios generally less than 1.2 for disorders and less than 1% of the variance explained for continuous traits.

Although known GWA results only explain about 5% of the heritability of complex traits, it is at least theoretically possible that common variants of smaller effect size in the population can account for heritability (Gibson, 2010). Some empirical support for this possibility has also been reported: A novel analysis of height suggests that common variants might be able to account for nearly all of the heritability of height (Yang et al., 2010); similar results have been reported for common disorders (Lee, Wray, Goddard, & Visscher, 2011).

In addition to increasing sample sizes in order to detect associations with common variants of very small effect size, several other strategies could increase power to detect such small effects. GWA studies use a conservative threshold for significance that corrects for genome-wide multiple testing (usually *p* < 5 × 10^−8^), even though only relatively large effect sizes can clear such a high hurdle. Rather than increasing the sample size for a single definitive study, another way to increase power to detect small effects is to use a series of replication studies with less conservative *p* values in order to winnow the small kernels of grain from the chaff, side-stepping some of the daunting issues involved in correcting for multiple testing in a single GWA study (Davis et al., 2010; Docherty, Davis, et al., 2010).

Another promising direction is to focus on polymorphisms that are known to be functional, that is, polymorphisms whose alleles are known to make a difference phenotypically (Cooper et al., 2010). Functional polymorphisms add considerable power in GWA research by testing a direct association between the polymorphism and the trait rather than relying on indirect association via neighboring polymorphisms. For example, a microarray is available that includes 15,000 SNPs in coding genes that result in a change of an amino acid during translation (Birney et al., 2007). Another strategy for identifying functional polymorphisms is to identify associated differences in gene expression profiles throughout the genome (Pickrell et al., 2010). In addition, research is underway to catalog functional polymorphisms in noncoding regions of the genome (Alexander, Fang, Rozowsky, Snyder, & Gerstein, 2010). Eventually, whole-genome sequencing will make it possible to examine polymorphisms of any kind.

A final example of strategies for increasing the power to detect associations of small effect is to go beyond a SNP-by-SNP analysis to a gene-by-gene analysis or multiple-gene systems in functionally related groups of genes. For example, a study of cognitive development in children reported an association with a composite of SNPs composed from genes in a functional gene group involving G proteins which are important in cellular signaling (Ruano et al., 2010).

**Figure 1:**
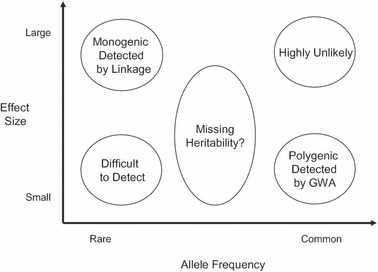
The relation between effect size and allele frequency for detecting associations. *Note.* GWA = genome-wide association. Adapted from McCarthy et al. (2008).

Despite these efforts, it seems unlikely that common variants with very small effects will completely solve the missing heritability problem. Figure 1 frames the issue by plotting allele frequency (from small to large) against effect size (from rare to common). As indicated in the lower right-hand corner, GWA can detect associations with common alleles (population frequencies greater than 1%) unless the effect sizes are miniscule. As indicated in the upper left-hand corner, linkage analysis can detect rare alleles with large effects. The upper right-hand corner (common alleles with large effect) seldom occurs. The most daunting prospect is the lower left-hand corner: very rare alleles of small effect, which will be extremely difficult to detect. In between these extremes, in the middle of Figure 1, is a promising area for finding missing heritability: less common alleles (< 1%) not tagged by current microarrays yet common enough to show modest effects in the population. New microarrays are being designed to capture these variants and whole-genome sequencing will eventually detect all variants no matter how rare. It is likely that all five circles in Figure 1 contribute to the missing heritability.

### Rare Variants Could Have Large Effects

We suggest that a good target for missing heritability is the middle of Figure 1—variants that are not rare but are less common than those tagged by commercially available microarrays. However, the missing heritability argument tends to be phrased in terms of common variants of small effect (lower-right circle) versus rare variants of large effect (upper-left circle; Goldstein, 2009; Hirschhorn, 2009; McClellan & King, 2010; Schork, Murray, Frazer, & Topol, 2009). Although 90% of human DNA variation involves evolutionarily old mutations that have filtered through the population and are now relatively common (McClellan & King, 2010), it has been estimated that we each harbor 175 new mutations, many of which will be found in only one person or one family (Nachman & Crowell, 2000). Many of these new mutations have no functional significance and thus persist in the population in the absence of selective pressure. However, mutations with large effects are likely to be deleterious (because the existing genome has been fine-tuned by evolution) and result in a decrease in reproductive fitness, so that mutations with large effects are likely to be of recent origin and therefore rare.

The rare-variant-of-large-effect position gained strength as the first harvest of GWA studies using common variants exposed the missing heritability problem (Bodmer & Bonilla, 2008; Cirulli & Goldstein, 2010; Galvan, Ioannidis, & Dragani, 2010; Gorlov, Gorlova, Sunyaev, Spitz, & Amos, 2008; Schork et al., 2009). The idea is that common diseases could be due to rare deleterious variants that have a strong impact on the risk of disease in individual patients but would not be detected by linkage across different families (Risch & Merikangas, 1996), although studies of single large pedigrees might be able to detect them (Galvan et al., 2010). It is possible that the weak associations found for common variants might reflect indirect associations between these common variants and rare genetic variants of large effect (Dickson, Wang, Krantz, Hakonarson, & Goldstein, 2010), although this has been disputed (Wray, Purcell, & Visscher, 2011).

Whole-genome sequencing of large samples as in the 1000 Genomes Project mentioned earlier is expected to identify millions of new rare SNPs and CNVs, most of which are likely be new mutations specific to an individual (Kuehn, 2008). This will raise new design and analytic issues because each of us may have hundreds of thousands of rare variants (McClellan & King, 2010). For rare variants of large effect, one possibility is to go back full circle to linkage studies of a single large pedigree with the mutation (Bourgain, Genin, Cox, & Clerget-Darpoux, 2007). For new mutations of small effect (lower left circle in Figure 1), it may be possible to use a polygenic composite that sums the number of such mutations across the genome as an overall index of genetic risk, because most such mutations are likely to be deleterious (Li & Leal, 2008; Morris & Zeggini, 2010).

The rare-variant position was enhanced by the discovery of CNVs, described earlier, which have been shown to contribute to several disorders (Mefford & Eichler, 2009; Stankiewicz & Lupski, 2010; Wain, Armour, & Tobin, 2009), especially schizophrenia (Sebat, Levy, & McCarthy, 2009; Stefansson et al., 2008; Williams, Owen, & O’Donovan, 2009), autism (Bucan et al., 2009; Glessner et al., 2009), and most recently hyperactivity (Williams et al., 2010). The initial excitement about CNVs as a solution to the missing heritability problem has dulled somewhat for two reasons. First, CNVs are difficult to detect reliably using available microarrays; moreover, most common CNVs are tagged reasonably well by SNPs on existing microarrays, which implies that common CNVs are unlikely to fill in much of the missing heritability (Craddock et al., 2010). However, most CNVs are not common (Itsara et al., 2009), which leaves CNVs as a contender for rare-variant explanations of missing heritability. Similar to any DNA variant, CNVs with large effects are likely to be rare because they ought to be subjected to negative selection.

The second reason is that CNVs associated with autism and schizophrenia appear to be de novo, that is, newly formed mutations that are not inherited from either parent (Awadalla et al., 2010; Stefansson et al., 2008; Stone et al., 2008; Weiss et al., 2008). If mutations are not inherited they are not a source of missing heritability.

### Other Sources of Missing Heritability: Epistasis, Gene–Environment Interaction, Epigenetics

Although most discussion of the missing heritability problem has focused on the dimension of rare versus common variants, many other possible sources of missing heritability have been mooted. We will briefly discuss three other possible sources of missing heritability. Most often mentioned is the possibility of interactions—interactions between genes (called *epistasis*) or interactions between genes and environment. These are daunting possibilities because if it is so difficult to detect “main effect” associations between DNA variants and behavioral traits, it will be much more difficult to detect interactions in which the association with a gene depends on other genes or on specific environments.

For both gene–gene and gene–environment interaction, it is important to distinguish different connotations of the word *interaction* (Kendler & Gardner, 2010). At the level of processes and mechanisms, interaction is crucial. However, in quantitative genetics, the word *interaction* is used much more narrowly to refer to a statistical interaction in the sense of analysis of variance; this is the context in which heritability is estimated. In this narrow quantitative genetic context, heritability refers to the “main effect” of genetic differences on individual differences in behavior. Translated to molecular genetics, heritability denotes the extent to which DNA variants are associated with individual differences in traits. The issue of interaction involves the extent to which these associations depend on—are moderated by—other DNA variants or by environmental conditions.

It has been suggested that gene–gene interaction is widespread and could contribute importantly to missing heritability because GWA studies generally identify only additive effects of each gene (Cordell, 2009; Moore & Williams, 2009; Phillips, 2008). However, there are reasons to doubt the importance of epistatsis (Cirulli & Goldstein, 2010). Although animal model research has found some examples of epistasis (Valdar et al., 2006), these examples usually involve major-gene pathological mutations. In contrast, most complex traits including behavioral traits show additive genetic effects (Flint, DeFries, & Henderson, 2004). Evolutionary reasons why genetic effects are likely to be additive have also been mooted (Hill, Goddard, & Visscher, 2008).

Although molecular genetic data will be needed to determine definitively the importance of epistasis, quantitative genetic research provides some support for the view that most genetic effects are additive. Quantitative genetic estimates of heritability discriminate between *narrow heritability,* which is limited to additive genetic variance, and *broad heritability*, which also includes nonadditive (epistatic) genetic variance (Plomin et al., 2008). For example, parents and offspring only share additive genetic effects so that estimates of heritability based on parent–offspring designs estimate narrow heritability. In contrast, the twin design, which compares MZ and DZ twin similarity, estimates broad heritability (see Plomin et al., 2008, for details). Comparing estimates of narrow and broad heritability does not suggest much evidence for epistatic effects. Nonetheless, it seems likely that gene–gene interaction will identify some of the missing heritability.

Similarly, although gene–environment interaction is the crucial interface for understanding mechanisms by which genes have their effect on development (Rutter, 2007), the limited question here is the extent to which gene–environment interaction accounts for heritability in the quantitative genetic sense of associations between DNA variants that are moderated by environmental factors. Similar to gene–gene interaction, the potential effect of gene–environment interaction on estimates of heritability depends on the particular quantitative genetic design (Plomin et al., 2008). Much has been written about gene–environment interaction; the human and nonhuman quantitative genetic literature suggests that gene–environment interaction is also a viable candidate to explain some of the missing heritability.

In addition to epistasis and gene–environment interaction, gene expression in general and epigenetics in particular have been mentioned in relation to missing heritability. The double-helix structure of DNA evolved as a mechanism for heritable transmission from generation to generation. Single-stranded RNA evolved to transcribe the DNA code in response to the environment; this is the process of gene expression. Gene expression changes from second to second—as you read this, the transcription of DNA to RNA is changing for many genes in your brain. Epigenetic mechanisms, principally alterations in methylation of DNA and changes in its chromatin structure, silence gene expression and result in slow-motion, developmentally stable changes in gene expression without altering DNA sequence (Jaenisch & Bird, 2003). Just as microarrays have made it possible to conduct DNA analysis across the entire genome, microarrays have also made it possible to study profiles of gene expression (as assessed by RNA transcripts) transcribed from all coding genes in the genome (called the *transcriptome*) and profiles of DNA methylation of all coding genes in the genome (called the *methylome* or *epigenome*). Because gene expression and methylation were designed by evolution to be sensitive to the environment, the transcriptome and epigenome could be useful as biomarkers of environmental change (Jirtle & Skinner, 2007; Mill & Petronis, 2008; Mill et al., 2008; Petronis, 2010), including prenatal experiences (Zhang & Meaney, 2010) and mother–infant interaction (Champagne & Curley, 2009; Meaney, 2010).

How does this relate to the issue of missing heritability? The short answer is “not much” (Slatkin, 2009). Although epigenetics is often defined as heritable changes in gene expression, the use of the word *heritable* is confusing because in the case of epigenetics it only refers to stable changes in gene expression as cells divide during mitosis, which is very different from the normal use of the word *heritable*, which denotes inheritance of DNA variation from generation to generation. Individual differences in DNA methylation are best considered as a phenotypic trait, which could be due to genetic as well as environmental differences. Indeed, individual differences in DNA methylation across the genome appear to be largely environmental in origin, as indicated by classical twin studies that compare MZ and DZ twins (Kaminsky et al., 2009; Wong et al., 2010).

Nonetheless, unlike other mechanisms of gene expression, some epigenetic effects can be transmitted across generations through a process known as genomic imprinting, which could affect heritability estimates (Daxinger & Whitelaw, 2010; Nelson & Nadeau, 2010). Genomic imprinting is a process by which certain genes are expressed in a parent-of-origin-specific manner, that is, in which alleles are only expressed when inherited from the mother or from the father (Tollkuhn, Xu, & Shah, 2010). Although only a few dozen genes have been shown to be classically imprinted in humans, it is possible that subtle imprinting effects could be more widespread (Schalkwyk et al., 2010); for nonhuman mammals, recent research has extended the list of imprinted genes from about 100 to more than 1,000 (Gregg, Zhang, Butler, Haig, & Dulac, 2010; Gregg, Zhang, Weissbourd, et al., 2010). Moreover, some SNP associations have been shown to be moderated by parent-of-origin effects resulting in stronger associations with one parent or the other (Kong et al., 2009). This dilution of SNP associations when parent-of-origin effects are ignored in GWA studies could contribute to missing heritability (Schalkwyk et al., 2010). In addition, if parent-of-origin effects are widespread, they might inflate heritability estimates if MZ twins, who derive from the same fertilized egg, share such imprinting effects to a greater extent than DZ twins. However, caution has been advised in this fast-growing field of behavioral epigenetics (Buchen, 2010), especially in relation to human behavior (Miller, 2010).

## Implications for Developmentalists

The goal of the 1998 article was to begin a discussion about “what to do with genes once they are found.” I continue to believe that once genes are found they will transform the ability of developmental research to address questions about developmental continuities, about psychopathological patterns, and about environmental risk mechanisms. The present article described two technological advances since 1998 that will facilitate the use of DNA in developmental research. The first is the microarray, which makes it possible to go beyond a few candidate genes to study thousands of DNA variants relevant to the development of behavioral domains as they interact and correlate with the environment. The second is a longer term prospect: If the predictions described earlier come true that whole-genome sequencing will be routinely obtained for newborns for purposes of genomic screening and personalized medicine, then all DNA variation throughout the genome could be available potentially for use in research without conducting any genotyping and even without collecting DNA.

The present article attempted to update the 1998 article, especially to consider a crucial problem that has emerged since 1998: the slow progress in identifying the genes responsible for the heritability of quantitative traits and common disorders. The main implication of GWA studies is that the missing heritability is likely to be due to many DNA variants of small effect; although rare variants of large effect exist, they are unlikely to be a major source of heritability in the population (Wray et al., 2011). Two practical implications follow from the conclusion that heritability is due to many DNA variants of small effect. First, it will be difficult to identify DNA associations of very small effect size and it will be even more difficult to replicate such associations. Second, as these DNA variants are found, their application to developmental research will require highly polygenic approaches involving hundreds or thousands of genes. Fortunately, custom microarrays that genotype thousands of DNA variants are less expensive than genotyping a few DNA variants individually. Moreover, if whole-genome sequencing becomes widely available it will entirely sidestep the issue of cost, as indicated earlier.

A third implication of the conclusion that heritability is due to many genes of small effect is more conceptual and far reaching, especially for developmental psychopathology. To the extent that heritability is due to alleles of small effect, regardless of how common or rare the alleles are, it supports the basic quantitative genetic model as developed by Fisher in 1918. Fisher reconciled the two worlds of genetics—the monogenic world of Mendel’s newly rediscovered work on single-gene effects that led to qualitative disorders and the polygenic world of Galton that led to quantitative traits. The essence of Fisher’s insight was that genes could work hereditarily as Mendel’s experiments indicated, but if a trait is influenced by several genes it would be normally distributed as a quantitative trait. The converse of this insight is important in the present context: If a disorder is influenced by many genes—and this is the conclusion that emerges from GWA research—its genetic liability is likely to be normally distributed. Thus, in terms of genetic liability, common disorders are quantitative traits (Plomin, Haworth, & Davis, 2009). In other words, genes that are found to be associated with disorders in case-control studies are predicted to be correlated with the entire range of variation throughout the normal distribution. Stated more provocatively, this means that from a genetic perspective there are no common disorders, just the extremes of quantitative traits.

An important caveat is that common disorders are genetically heterogeneous and often include versions of the disorder caused by a rare variant of major effect. For example, 282 monogenic disorders include lowered IQ among their symptoms, such as the well-known disorders phenylketonuria and fragile X syndrome, as well as many disorders less known in this context such as neurofibromatosis and Duchenne muscular dystrophy (Inlow & Restifo, 2004). However, because these disorders are so rare in the population, these genes contribute little to the heritability of IQ in the population despite their dramatic impact on affected individuals. That is, most of the heritability is due to genes of small effect.

Three important implications follow from this conclusion that common disorders are quantitative traits. First, at all levels of analysis from biology to brain to behavior, it should stimulate research on quantitative dimensions rather than qualitative disorders. Although the extremes of these quantitative dimensions are important medically and socially, there seems to be no scientific advantage in reifying diagnostic constructs that have evolved historically on the basis of symptoms rather than etiology. Focusing on quantitative dimensions could lead to a new approach to psychopathology based on etiology rather than symptoms (Plomin et al., 2009).

Second, research on quantitative dimensions leads away from the notion of curing diagnosed “cases” toward a public health model that focuses on preventing problems and promoting health rather than curing illness once it occurs (Brownson, Fielding, & Maylahn, 2009). The third implication is related but warrants separate consideration. Because polygenetic liabilities are normally distributed as a bell-shaped curve, they draw attention to the neglected positive side of the liability in addition to the negative side that has been the focus of most genetic research to date. The positive side of polygenic liability is not just low risk: It stimulates questions such as how children flourish rather than fail and about resilience rather than vulnerability. These are themes that characterize the field of positive psychology (Seligman & Csikszentmihalyi, 2000). For this reason, this new direction for genetic research has been called *positive genetics* (Plomin et al., 2009).

As the dust begins to settle from the explosions from the new advances in molecular genetics such as microarrays, genome-wide sequencing, and GWAs studies, this article ends with an appeal for open-mindedness and tolerance. Because molecular genetics is such a fast-moving area of research, there is a flavor-of-the-month faddism with favor passing from linkage to candidate gene to GWA approaches, from common variants to rare variants, and from microarrays to whole-genome sequencing. Is GWA the panacea for finding genes? No. Is it useful? Definitely. In the search for the effects of rare variants, a more balanced research portfolio seems warranted that includes linkage and candidate-gene (functional) approaches. The answer to the current debate about common versus rare variants is that both are likely to be important. Finally, the field is currently waiting for the next big thing, whole-genome sequencing. Will it be a panacea? No. Will it be useful? Definitely.
